# Japanese clinical guidelines for neovascular age-related macular degeneration

**DOI:** 10.1007/s10384-025-01240-0

**Published:** 2025-07-14

**Authors:** Tomohiro Iida, Fumi Gomi, Tsutomu Yasukawa, Kenji Yamashiro, Shigeru Honda, Ichiro Maruko, Keiko Kataoka, The Japanese Retina and Vitreous Society Guidelines Committee for Neovascular Age-Related Macular Degeneration

**Affiliations:** 1 Department of Ophthalmology, Tokyo Women’s Medical University, 8-1 Kawadacho, Shinjuku-ku, Tokyo Japan; 2 Department of Ophthalmology, Ageo Central General Hospital, Ageo, Japan; 3 Department of Ophthalmology, Hyogo Medical University, Nishinomiya, Japan; 4 Department of Ophthalmology and Visual Science, Nagoya City University Graduate School of Medical Sciences, Nagoya, Japan; 5 Department of Ophthalmology and Visual Science, Kochi Medical School, Kochi University, Kochi, Japan; 6 Department of Ophthalmology and Visual Sciences, Osaka Metropolitan University Graduate School of Medicine, Osaka, Japan; 7 Department of Ophthalmology, Kyorin University School of Medicine, Tokyo, Japan

**Keywords:** Neovascular AMD, Guideline, Pachychoroid, Polypoidal choroidal vasculopathy, Macular neovascularization

## Abstract

Recent advances in imaging technology and increased options of pharmaceutical therapy require that guidelines on the diagnostic criteria and treatment of neovascular age-related macular degeneration (AMD) be updated at regular intervals. These guidelines aim to standardize the management of neovascular AMD based on the latest understanding of its pathophysiology, advancements in diagnostic imaging modalities, and treatment options. The key updates include: (1) a revision of terminology and stage classification, adopting the AMD classifications of atrophic and neovascular, and adding end-stage AMD to the existing early, intermediate, and late stages; (2) the inclusion of pachychoroid in addition to drusen in the initial pathophysiology and pathogenic background; (3) diagnostic criteria defined by the presence of macular neovascularization based on multimodal imaging, including optical coherence tomography (OCT) and OCT angiography; (4) assessment of disease activity based on OCT; and (5) treatment guidance, including prophylaxis and low vision care as well as loading and maintenance phases by use of anti-vascular endothelial growth factor therapy and adjunctive therapies. We hope that these guidelines will be useful for those working in clinical practice in Japan, in other Asian countries, and in countries outside Asia.

## Introduction

Age-related macular degeneration (AMD) is a refractory disease and a major cause of visual impairment in developed countries. The development of optical coherence tomography (OCT) in the 21st century has made it easier to diagnose and classify the disease types of neovascular AMD and to evaluate treatment effectiveness. In addition to the progress in diagnosis and classification, photodynamic therapy (PDT) and intravitreal injections of anti-vascular endothelial growth factor (anti-VEGF) drugs have significantly improved the visual outcomes of patients with neovascular AMD.

Guidelines should be updated periodically to deliver the latest information on diagnosis and treatment to ensure the quality of standard care. The recent development of optical coherence tomography angiography (OCTA) has enabled detailed analysis of neovascular AMD before starting treatment, and the emergence of new drugs has expanded the treatment options for AMD. Furthermore, the accumulated experience in treating AMD has taught us that some patients with neovascular AMD do not derive the expected benefits from treatment, especially at the end-stage. Pachychoroid is a new form of AMD, introduced in 2013, that is now widely accepted [1]. However, there are currently no guidelines for the management of AMD associated with pachychoroid. In Asian countries, polypoidal choroidal vasculopathy (PCV) is considered a major subtype of neovascular AMD, and some cases of PCV develop against a background of pachychoroid [2]. The first-line treatment for PCV has shifted to monotherapy with anti-VEGF drugs, rather than monotherapy or combination therapy with photodynamic therapy (PDT). Like PCV, neovascular diseases related to the pachychoroid have been managed in much the same way as neovascular AMD, both before and after the introduction of the pachychoroid concept. As such, pachychoroid-related pathophysiology and diseases should be included in guidelines for neovascular AMD.

**Fig. 1:**
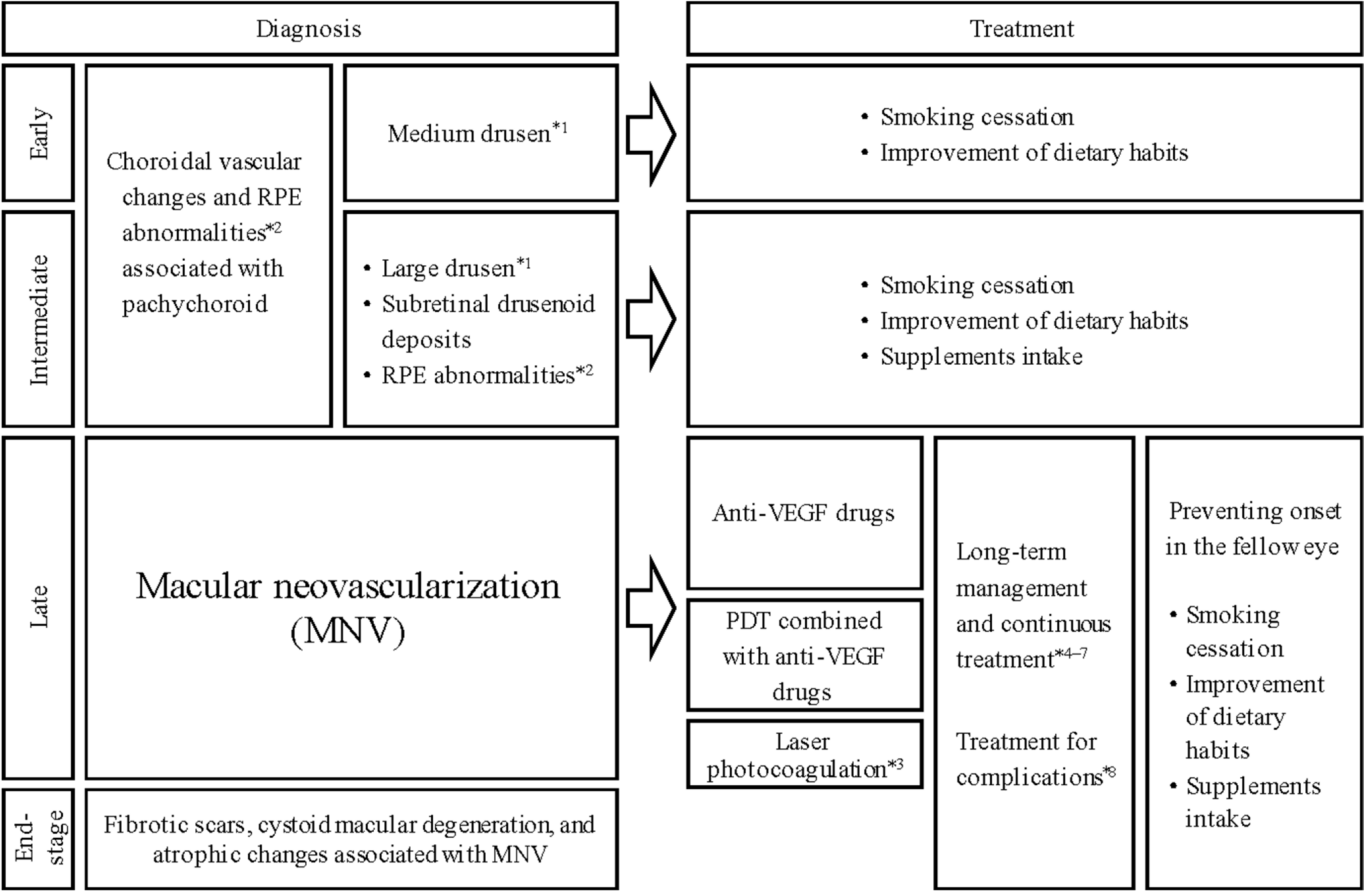
Diagnostic flowchart for neovascular age-related macular degeneration. *1: Medium drusen: ≥63 µm and <125 µm; large drusen: ≥125 µm. *2 Retinal pigment epithelial detachment (PED) without MNV.*3: Not suitable for MNV close to the fovea because of irreversible damage to the outer retina and RPE. *4 Treatment regimens include treat-and-extend, fixed dosing, or pro re nata (PRN). *5: For patients with inadequate or no response to treatment, consider switching anti-VEGF drugs or combining anti-VEGF therapy with PDT. *6 Observation of fibrotic scars with low disease activity, cystoid macular degeneration, and atrophy. *7: Provides low-vision care for patients with poor visual acuity in both eyes. *8 Submacular hemorrhage, vitreous hemorrhage, and endophthalmitis. RPE, retinal pigment epithelium; VEGF, vascular endothelial growth factor; PDT, photodynamic therapy

After collecting and discussing the evidence within the working group, we developed the new diagnostic criteria and treatment strategies of neovascular AMD. The updated guidelines include the pachychoroid for the first time. We also revised the disease stage classification and the treatment guidelines based on the latest evidence (Fig. 1). Importantly, AMD are sometimes seen even in patients under 50 years of age, and thus, age was removed from the updated diagnostic criteria.

## Revision of terminology and staging

### Classification of AMD

AMD is classified into “dry AMD” and “wet AMD.” The terms atrophic, non-exudative, and non-neovascular have also been used for dry AMD, whereas exudative and neovascular have been used for wet AMD. However, the presence of AMD with neovascular lesions but no exudative changes suggests that we should avoid using the terms: dry, wet, non-exudative, and exudative. Considering a report by the Neovascular AMD Nomenclature Study Group [3] we modified the classification of AMD into atrophic and neovascular AMD in the current guidelines.

### Macular neovascularization

Previously, the term choroidal neovascularization (CNV) was commonly used to describe the neovascularization observed in the macular region of neovascular AMD. However, as neovascularization can also originate from retinal vessels in the macular area, the term macular neovascularization (MNV) has been used internationally since 2020 [3]. Considering this, MNV is used in the current guidelines instead of CNV.

### Atrophic changes

Atrophic changes occur in both atrophic AMD and neovascular AMD. The clearly demarcated atrophic lesions involving the outer retina, retinal pigment epithelium (RPE), and choriocapillaris observed in atrophic AMD have long been referred to as geographic atrophy (GA) [4]. In contrast, similarly well-defined atrophic lesions can also be observed before and after treatment in neovascular AMD with MNV. To distinguish these atrophic lesions from GA, the term macular atrophy has increasingly been used. According to the international guidelines published in 2018, the term macular atrophy can be applied to well-defined atrophic lesions, regardless of the presence of MNV [5]. Since GA and macular atrophy are distinct from the atrophic changes in the outer retina associated with fibrotic scarring and cystoid macular degeneration observed in the end-stage of neovascular AMD, atrophic changes are the preferred term used in the current guidelines to define end-stage neovascular AMD.

### Stage classification

Several study groups have classified AMD into three stages based on the age-related eye disease study (AREDS): early, intermediate, and late [6]. However, the definitions for each stage are not consistent with the size, number, and location of drusen deposits and GA. Since drusen deposits and GA are relatively rare in Asians compared with Caucasians, the current guidelines classify AMD based on the Beckman classification [7]. In addition to the early, intermediate, and late stages, we also propose a new stage of end-stage AMD. End-stage AMD defines a patient who is unlikely to achieve significant functional and anatomical improvement despite treatment.

## Diagnostic criteria 1: initial pathophysiology and pathogenic background

Drusen and RPE abnormalities have traditionally been recognized as the initial pathophysiological and pathogenic background of neovascular AMD. However, the recent pachychoroid concept states that previously diagnosed neovascular AMD includes pachychoroid-related diseases and suggests that the pachychoroid is also the initial pathophysiology and pathogenetic background of neovascular AMD (Fig. 1). In addition to the initial pathophysiology and pathogenetic background, this section describes genetic polymorphisms that have been studied in recent years.

### Drusen

Drusen deposits are located beneath the RPE. Previously, drusen had been categorized into two types based on their appearance. Hard drusen have well-defined boundaries, while soft drusen have indistinct boundaries. Hard drusen deposits are typically smaller. In contrast, soft drusen deposits tend to be larger. Drusen deposits are commonly classified by size: those less than 63 µm are considered small, whereas 63 µm or larger but less than 125 µm are medium, and those 125 µm or larger are classified as large. Recently, the term hard drusen has been used for deposits smaller than 63 µm, and soft drusen for those 63 µm or larger. In the current guidelines, we also adopted a size-based classification. Note that the retinal vein diameter entering the optic disc is approximately 125 µm, serving as a reference point for determining drusen size.

A distinct entity similar to drusen are the subretinal drusenoid deposits, also known as reticular pseudodrusen or pseudodrusen. Subretinal drusenoid deposits are located above the RPE. Drusen and drusenoid deposits are widely recognized as risk factors for the development of neovascular and atrophic AMD [8].

### Pachychoroid

In Europe and the United States, drusen deposits are considered an important pathogenic factor in the development of AMD. However, drusen deposits are less common in Japanese patients with neovascular AMD (approximately 30%) [2, 9].

The concept of pachychoroid disease may be useful for explaining the pathogenesis of AMD despite the absence of drusen [10]. However, defining “pachychoroid” quantitatively is challenging. The term is often used when the choroid is thick considering age and refractive errors. Although the term pachychoroid initially referred to the finding of a thick choroid, it is widely thought that the term includes pathologic changes of the choroid such as pachyvessels (dilated large choroidal vessels) and the increased permeability of the choroidal vessels [10]. Pachychoroid is also being used generically to refer to these pathologic changes.

Central serous chorioretinopathy (CSC) is a common pachychoroid disease. In 2009, it was reported that, in patients with CSC the choroid is thick [11]. Conversely, in 2013, a disease entity labelled pachychoroid pigment epitheliopathy (PPE) was proposed for conditions involving pachychoroid with associated RPE abnormalities [1]. PPE is considered an incomplete form of CSC, referring to cases without coexisting nor pre-existing serous retinal detachment.

MNV can develop following CSC or PPE. When MNV occurs, it is referred to as pachychoroid neovasculopathy (PNV) [12]. In Japan, nearly half of the cases previously diagnosed as neovascular AMD are actually PNV [13–15]. These updated guidelines consider PNV as a form of neovascular AMD.

GA may occur in the context of pachychoroid [16]. Ongoing research is exploring the differences between GA associated with pachychoroid and GA associated with drusen.

### RPE abnormalities

RPE abnormalities include pigmentary changes in the macula, such as pigment loss, hyperpigmentation, pigmentary mottling, and serous retinal pigment epithelial detachment (PED) without MNV. Due to improvements in the detection accuracy of MNV, these guidelines consider the confirmation of MNV as a critical factor in diagnosing neovascular AMD; eyes with large PED without MNV are classified into intermediate AMD (see “Diagnostic Criteria 3: Staging”).

### Genetic variants

Genetic factors are significant in the development of AMD [17]. Notably, genes, such as *ARMS2/HTRA1* and *CFH,* are strongly implicated. Additionally, other susceptibility genes include genes associated with the complement system, such as *C2/CFB*, *C3*, and *CFI*; and genes involved in lipid metabolism, such as *APOE*, *CETP*, and *LIPC*; and *VEGFA*, *TGFBR1*, *TIMP3*, and *TNFRSF10A*. These genes have been confirmed to be involved in the development of AMD in the Japanese population as well [18]. Importantly, the *CFH* gene contributes not only to the pathogenesis of drusen, but also to pachychoroid, CSC, and PNV [19–21]. Prognosis can be predicted by examining the genotypes of patients with AMD, such as the development of the disease in the fellow eye [22, 23]. This highlights the importance of utilizing each patient’s genetic information in personalized medicine in the future.

## Diagnostic criteria 2: classification of disease type

### Diagnostic criteria

Neovascular AMD is defined by the presence of MNV in the macular area within a 6000 µm diameter centered on the fovea, in association with drusen, pachychoroid, or RPE abnormalities. Although confirmation of the presence of MNV is preferable for a definitive diagnosis of neovascular AMD, a diagnosis of neovascular AMD can be made if the presence of MNV is strongly suspected with sufficient certainty based on the presence of hemorrhagic changes or fibrotic scars. The exclusion criteria include idiopathic cases at a young age and other diseases that can cause MNV, i.e., high myopia, degenerative diseases such as angioid streaks, inflammatory diseases, infectious diseases, intraocular tumors, trauma.

### Classification of MNV

CNV classification has traditionally been based on fluorescein angiography (FA) and histopathology. On FA, the classic lesions demonstrate well-defined hyperfluorescence in early angiography, followed by progressive fluorescein leakage, while the occult lesions have indistinct and gradually ill-defined hyperfluorescence. Histopathologically, CNV located below the RPE are classified as type 1, while those extending beyond the RPE into the subretinal space are classified as type 2.

In the current guidelines, occult CNV and type 1 CNV are categorized as type 1 MNV, classic CNV and type 2 CNV as type 2 MNV, and MNV originating from the retinal vessels as type 3 MNV. Coexisting type 1 and 2 MNV are classified as mixed type 1 and 2 MNV (Table 1).[3] In type 1 MNV, the presence of polypoidal lesions may be observed at the ends of the MNV, which has traditionally been termed polypoidal choroidal vasculopathy (PCV). Neovascular AMD with type 3 MNV is known as retinal angiomatous proliferation (RAP).

**Table 1:** Classification of macular neovascularization (MNV)

Type 1 MNV without polypoidal lesion
Type 1 MNV with polypoidal lesion (PCV)
Type 2 MNV Mixed type 1 and type 2 MNV
Type 3 MNV(RAP)

*MNV* macular neovascularization, *PCV* polypoidal choroidal vasculopathy, *RAP* retinal angiomatous proliferation

Neovascular AMD characterized by drusen can present as type 1, 2, or 3 MNV. In contrast, pachychoroid diseases, such as PNV, primarily present as type 1 MNV.

## Diagnostic criteria 3: staging

These guidelines classify AMD into early, intermediate, late, and end stages. Although choroidal vascular changes and RPE abnormalities observed in eyes with the pachychoroid should be classified as either early or intermediate stages leading to PNV, no consensus has yet been reached to determine which stage of the disease matches the condition well.

### Early AMD

Early AMD is defined by the presence of medium-sized (soft) drusen (long diameter ≥ 63 µm and < 125 µm) in the macular area. Small (hard) drusen (long diameter < 63 µm) are not included in the criteria for early AMD. Instead, they are considered age-related changes within the physiological range. Although epidemiological data indicate that numerous (20 or more) or widespread small drusen increase the risk of developing medium-sized drusen and the risk of AMD onset [24], eyes with numerous small drusen are rarely seen in the Japanese population.

### Intermediate AMD

Intermediate AMD is defined by the presence of large (soft) drusen (long diameter ≥ 125 µm) or RPE abnormalities in the macular area. Subretinal drusenoid deposits are also classified as intermediate AMD, as they are widely recognized as risk factors for progression to late AMD, particularly type 3 MNV and atrophic AMD [8].

### Late AMD

All cases with MNV including PNV are classified as late AMD.

### End-stage AMD

Patients with severe visual loss owing to atrophic changes in the outer retina associated with fibrotic scars or cystoid macular degeneration are classified as having end-stage AMD.

## Diagnosis and activity assessment of neovascular AMD

The diagnosis of neovascular AMD and the assessment of the disease activity are made using visual acuity, ophthalmoscopic findings, fundus photographs, FA, indocyanine green angiography (ICGA), OCT, OCTA, and fundus autofluorescence (FAF) (Table 2). FA and ICGA (FA/ICGA) are not necessary when a diagnosis of neovascular AMD can be made based on other assessments. When considering FA and ICGA, it is necessary to keep in mind that these may lead to anaphylactic shock in susceptible patients.

**Table 2:** Image findings associated with neovascular age-related macular degeneration

	**Type 1 MNV**	**Type 2 MNV**	**Type 3 MNV**	**Polypoidal lesion**	**Characteristics related to pachychoroid diseases**
**Fundus examination**	Relatively flat retinal pigment epithelium elevation. Fibrinous exudate may be observed as a grayish-white elevated lesion.	A nodular white or yellowish-white elevated lesion. Fibrinous exudate may be observed as a grayish-white elevated lesion.	Angiomatous intraretinal neovascularization or anastomoses with retinal arterioles, venules, and choroidal vessels can be observed. Intraretinal hemorrhage, multiple drusen, subretinal drusenoid deposits are often observed.	Elevated orange-red lesion can be observed. Fibrinous exudate around polyps may be observed as a grayish-white elevated lesion.	Attenuated fundus tessellation due to the decreased visibility of large choroidal vessels. Pachydrusen are sometimes observed.
**OCT**	Relatively flat retinal pigment epithelial elevation (double layer sign) containing materials.	Subretinal or intraretinal materials through retinal pigment epithelium.	Materials in outer retina. Bump sign is often observed.	Protruded retinal pigment epithelial elevation.	Dilated larger choroidal vessels in Haller’s layer. Attenuated choriocapillaris and Sattler' layer.
	Contents are moderate or relatively high reflectivity.				
**OCTA**	MNV is observed on the *en face* image containing slab from retinal pigment epithelium to choriocapillaris.	MNV is observed on the *en face* image containing outer retinal slab.	MNV is difficult to observed on *en face* images.	Polypoidal lesion is difficult to observed on *en face* images.	–
	Abnormal blood flow signals are observed on B-scan images.				
**FA**	Early ill-defined hyperfluorescence and mild leakage in late phase.	Early well-defined and uniform hyperfluorescence and progressive leakage in late phase.	Retinal vascular anastomosis in early phase and progressive leakage and pooling of fluorescein in late phase.	Polypoidal lesion is sometimes observed as well-defined round hyprefluorescence in early phase. In late phase, polypoidal lesion often shows leakage.	–
**ICGA**	Lace-like hyperfluorescence in early phase and focal or plaque-like hyperfluorescent area in late phase.	Lace-like hyperfluorescence in early phase and focal or plaque-like hyperfluorescent area in late phase.	Retinal anastomosis in early phase and hot spot in late phase.	Dilated vessels are observed as well-defined nodular and round hyperfluorescence adjacent to plaque-like hyperfluorescence indicating type 1 MNV.	Dilated large choroidal vessels. Choroidal hyperpermeability is often observed in mid-phase of angiogram.

*OCT* optical coherence tomography, *OCTA* optical coherence tomography angiography, *FA* fluorescein angiography, *ICGA* indocyanine green angiography, *MNV* macular neovascularization.

### Diagnosis of MNV

#### Type 1 MNV

**Fig. 2:**
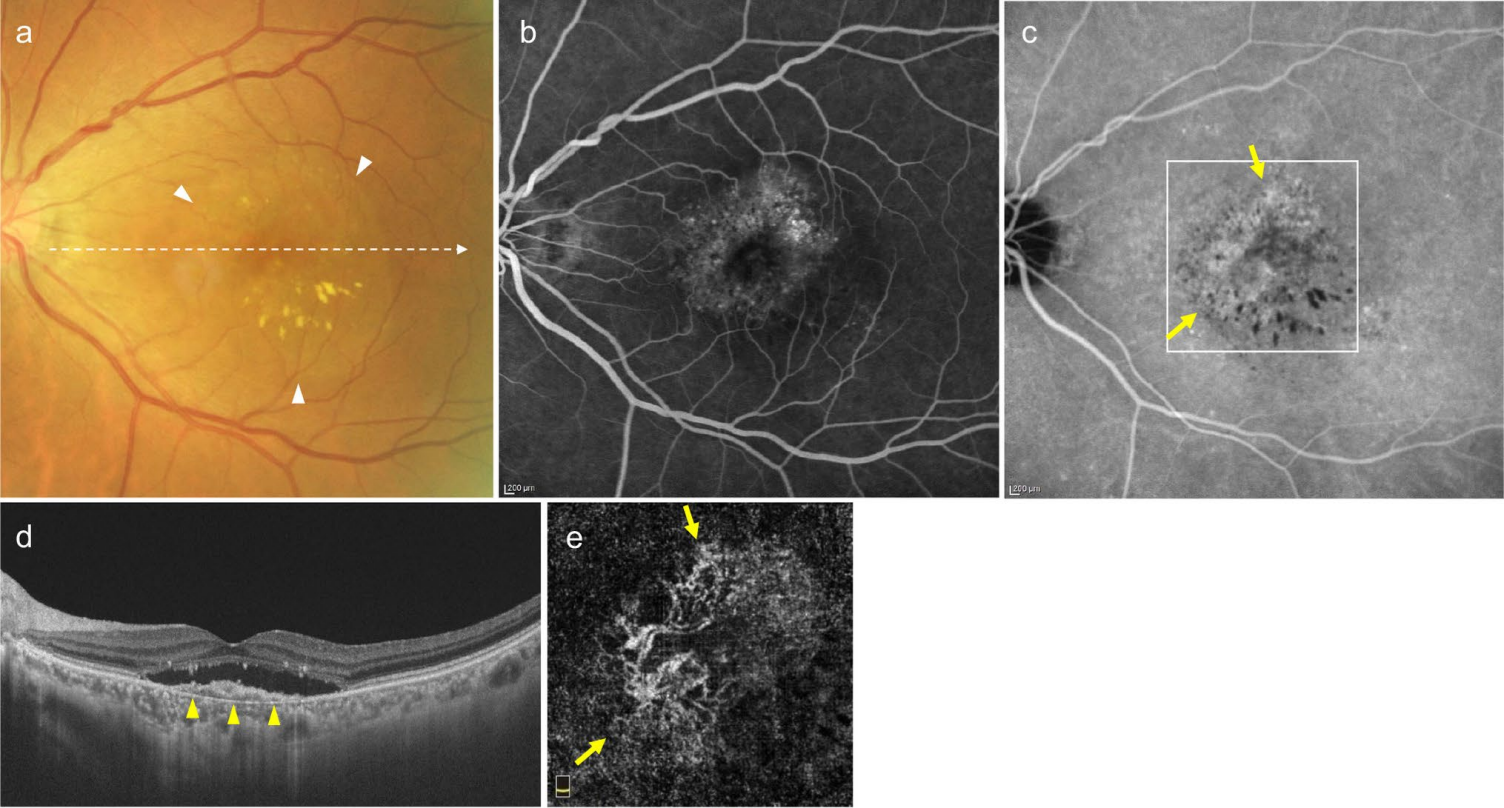
Type 1 macular neovascularization (MNV). **a** Hard exudates and serous retinal detachment (arrowhead) are observed in the fundus. **b** Late-phase fluorescein angiography (FA) shows mild leakage of fluorescence. Scale bar, 200 μm. **c** Indocyanine green angiography (ICGA) shows hyperfluorescence indicative of MNV (arrow). Scale bar, 200 μm. **d** Optical coherence tomography (OCT) corresponding to the dashed arrow in A shows subretinal fluid and a shallow elevation of the retinal pigment epithelium (RPE) with a double layer sign (arrowhead). **e** The en face OCT angiography (OCTA) image from the outer retina to the choriocapillaris, corresponding to the area indicated by the white square in C, shows MNV (arrow)

Type 1 MNV can be observed as relatively flat RPE elevations on ophthalmoscopy. However, its presence is more reliably determined using OCT, OCTA, or FA/ICGA (Fig. 2). The OCT findings, which appear as a double line of a low elevation of RPE and Bruch's membrane separated by type 1 MNV, are called the double-layer sign and are useful for suspecting type 1 MNV (Fig. 2D) [25]. OCTA is effective in visualizing MNV in *en face* images from the RPE to the choriocapillaris layer (Fig. 2E). Blood flow signals can also be confirmed under the RPE in B-scan images. In FA, type 1 MNV often shows ill-defined hyperfluorescence in the early phase, with gradual leakage in the late phase. The MNV presentation in ICGA is similar to *en face* OCTA images in the early phase, and hyperfluorescent areas in the late phase. Notably, early-stage type 1 MNV may be difficult to differentiate using FA/ICGA or OCT. However, OCTA sometimes provides higher detection sensitivity.

**Fig. 3:**
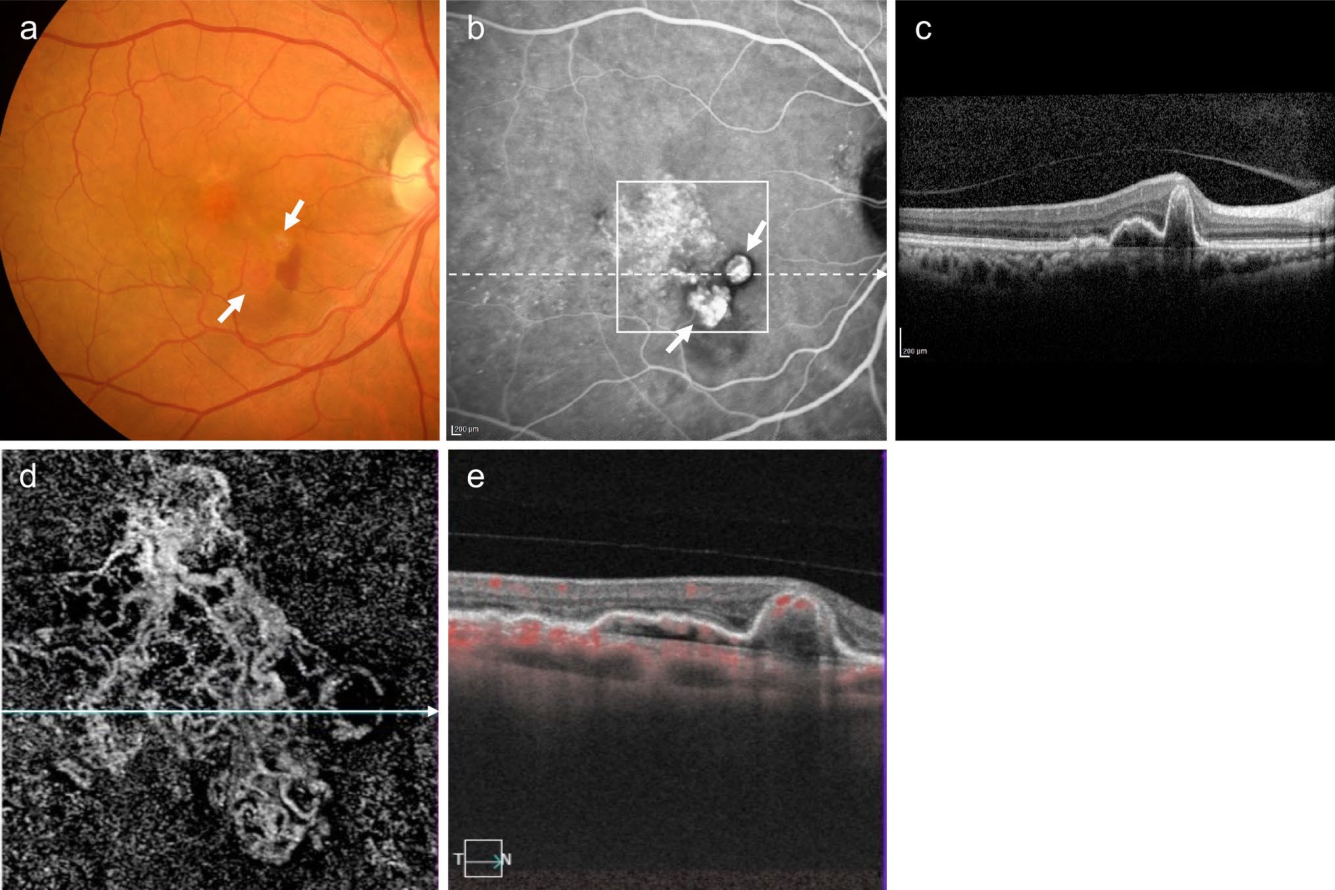
Polypoidal choroidal vasculopathy. **a** Fundus photo shows orange-red elevated lesions with subretinal hemorrhage (arrow). **b** ICGA shows nodular polypoidal lesions (arrow) continuous with type 1 MNV. Scale bar, 200 μm. **c** OCT corresponding to the site indicated by the dashed arrow in b shows a steep elevation of the RPE. Scale bar, 200 μm. **d** The en face OCTA image from the outer retina to the choriocapillaris, corresponding to the area indicated by the white square in b, shows MNV. **e** B-scan OCTA image corresponding to the site indicated in d shows blood flow signals (red) within the PED

**Fig. 4:**
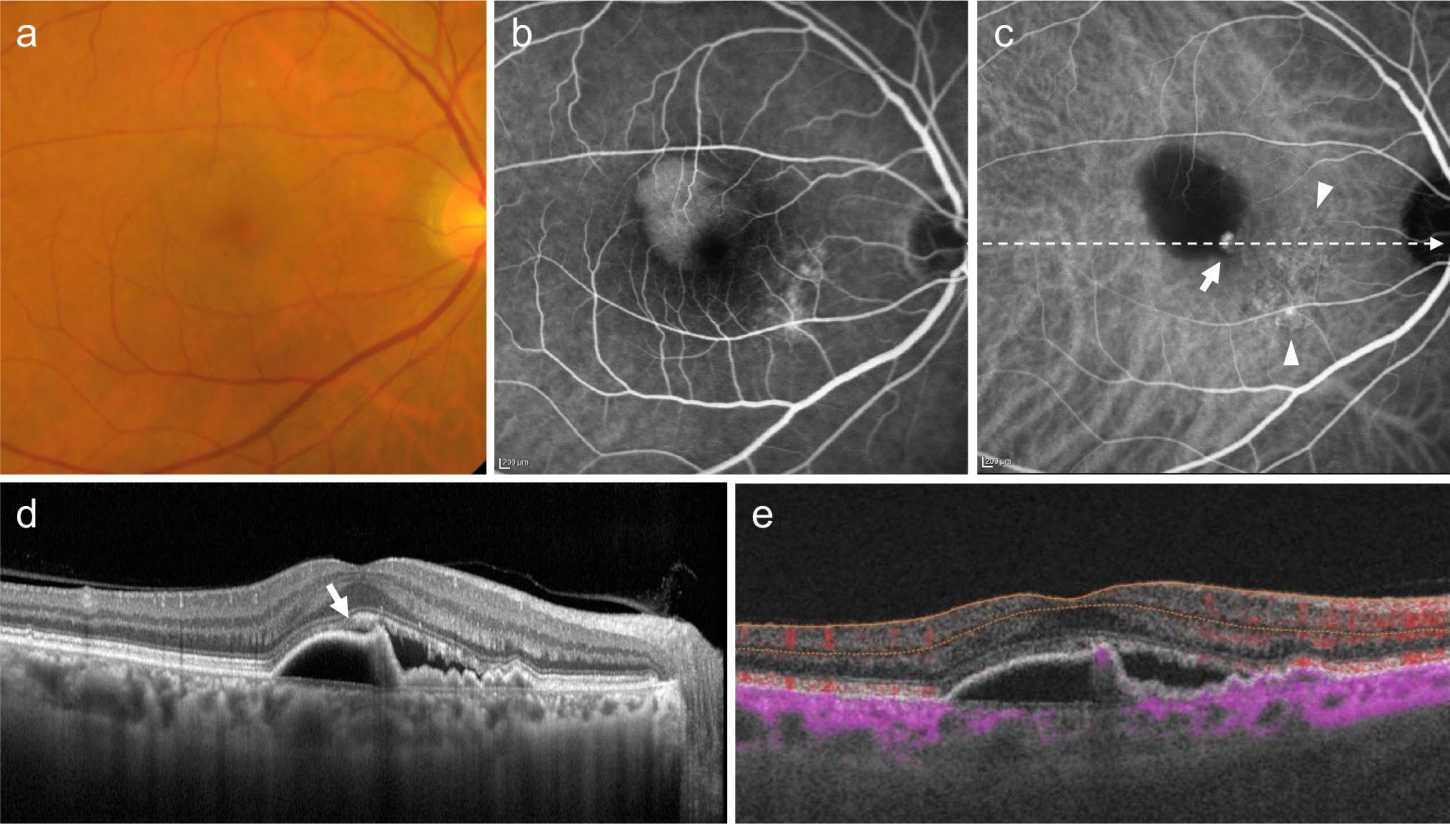
Polypoidal choroidal vasculopathy. **a** Fundus photo shows round PED. **b** FA shows dye pooling in the area of PED. Scale bar, 200 μm. **c** ICGA shows PED as a fluorescence block, with type 1 MNV (arrowhead) and polypoidal lesions (arrow). Scale bar, 200 μm. **d** OCT corresponding to the site indicated by the dashed arrow in c shows a serous PED with a notch (arrow) and a shallow elevation of the RPE with a double layer sign. **e** B-scan OCTA image shows blood flow signals (pink) at the site of the notch

**Fig. 5:**
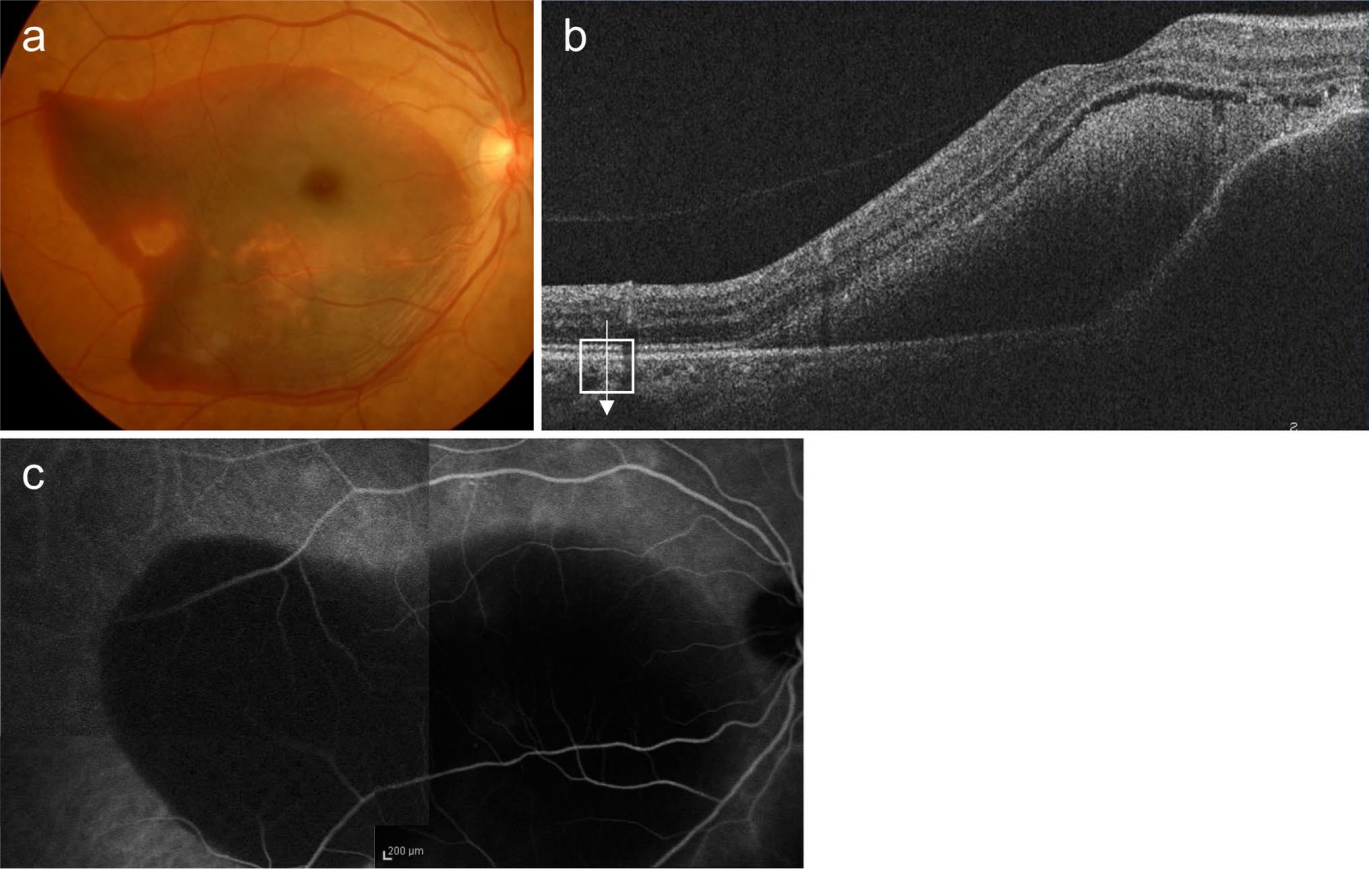
Massive subretinal and sub-RPE hemorrhage. **a** Fundus photo shows massive subretinal hemorrhage. **b** OCT shows hyperreflective subretinal hemorrhage and PED. **c** ICGA does not show MNV due to fluorescence blockage by hemorrhage. Scale bar, 200 μm

PCV is diagnosed when polypoidal lesions are confirmed. Polypoidal lesions can sometimes be observed as elevated orange-red lesions on ophthalmoscopy or fundus photography. However, they are more easily detected using OCT or ICGA (Fig. 3). On OCT, these lesions appear as protruding RPE elevations with moderate to high hyperreflectivity. Polypoidal lesions present with round, well-defined, and nodular dilated vascular lesions around type 1 MNV (previously called branching vascular networks) on ICGA. Notches in serous PED often correspond with type 1 MNV or polypoidal lesions (Fig. 4). A non-invasive diagnostic method for PCV has also been developed using OCT or OCTA [26–28]. Polypoidal lesions are characterized by sharply peaked PED and a sub-RPE ring-like lesion on OCT or flow signals on B-scan OCTA overlaid with round or ring-like OCT structures at the PED notch. PCV tends to cause bleeding and in the presence of massive subretinal or sub-RPE hemorrhage (hemorrhagic PED), polypoidal lesion detection can be challenging (Fig. 5).

#### Type 2 MNV

**Fig. 6:**
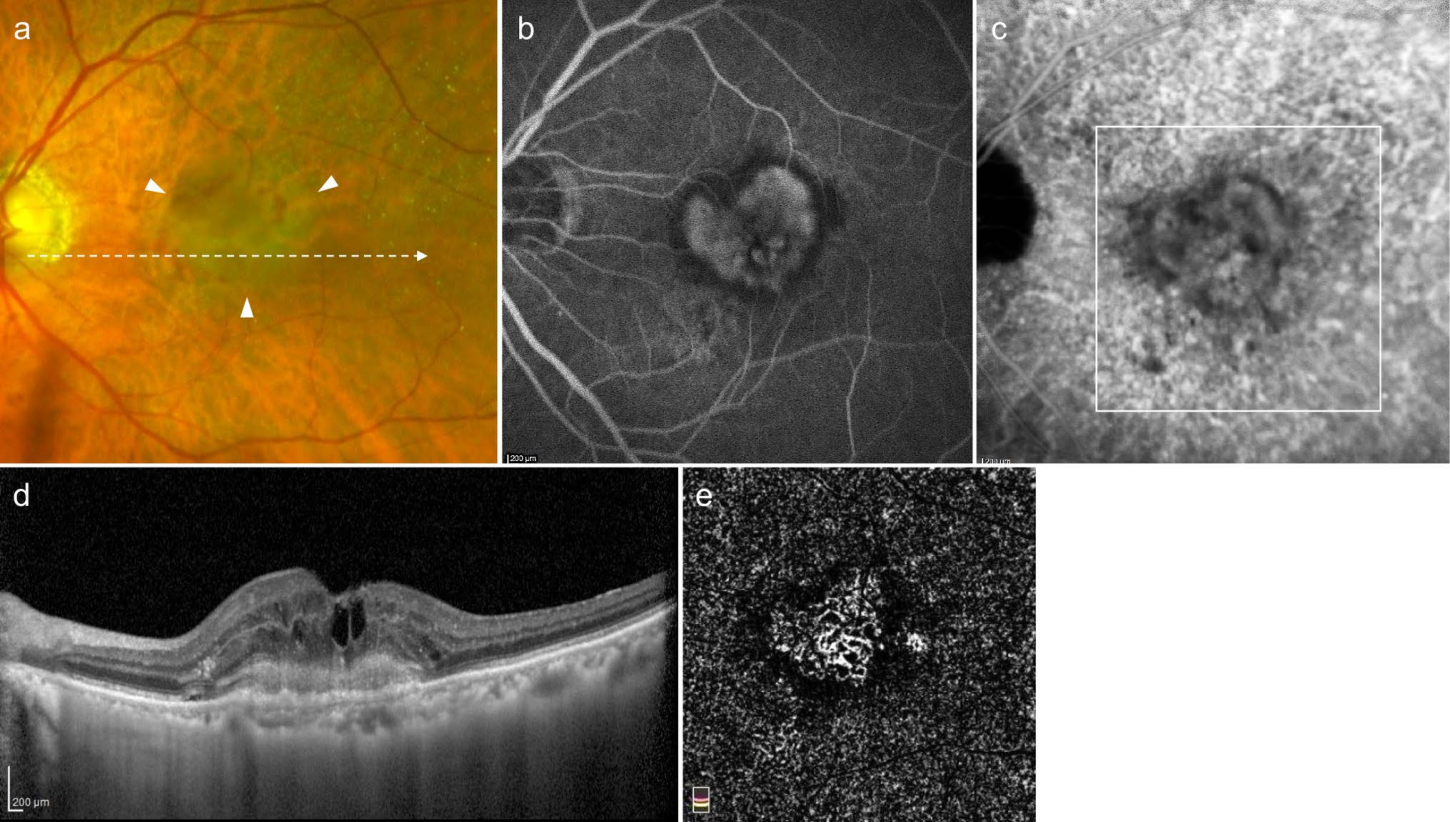
Type 2 MNV. **a** Fundus photo shows a gray-white lesion with hemorrhage and fibrinous exudates (arrowhead). **b**, **c** Early-phase FA (b) and late-phase ICGA (c) show hyperfluorescence indicating MNV. **d** OCT corresponding to the dashed arrow in (a) shows subretinal hyperreflective material under the retina. Scale bar, 200 μm. **e** The en face OCTA image from the outer retina to the choriocapillaris, corresponding to the area indicated by the white square in c, shows MNV

Type 2 MNV is often accompanied by a fibrinous exudate, appearing as a subretinal grayish-white elevated lesion on fundus examination. Subretinal hemorrhages can outline these elevations. Similar to type 1 MNV, type 2 MNV is more easily identified using OCT, OCTA, FA, and ICGA (Fig. 6). OCT shows moderate to high hyperreflective structures that extend beyond the RPE into the subretinal space or intraretina. However, as the disease activity decreases and the MNV become encapsulated in the RPE, the detection of type 2 MNV above the RPE becomes challenging. Furthermore, differentiating between subretinal fibrin and type 2 MNV using standard OCT is also challenging. By detecting blood flow signals in B-scan images, OCTA can be used to differentiate between subretinal fibrin and type 2 MNV. The two-dimensional morphology of type 2 MNV is observed in the *en face* OCTA images of the outer retinal layer (Fig. 6E). FA demonstrates well-defined MNV in the early phase, with progressive leakage in the late phase. In the early phase, ICGA presents similarly to OCTA *en face* images. In the late phase, plaque-like hyperfluorescence is evident.

#### Type 3 MNV

**Fig. 7:**
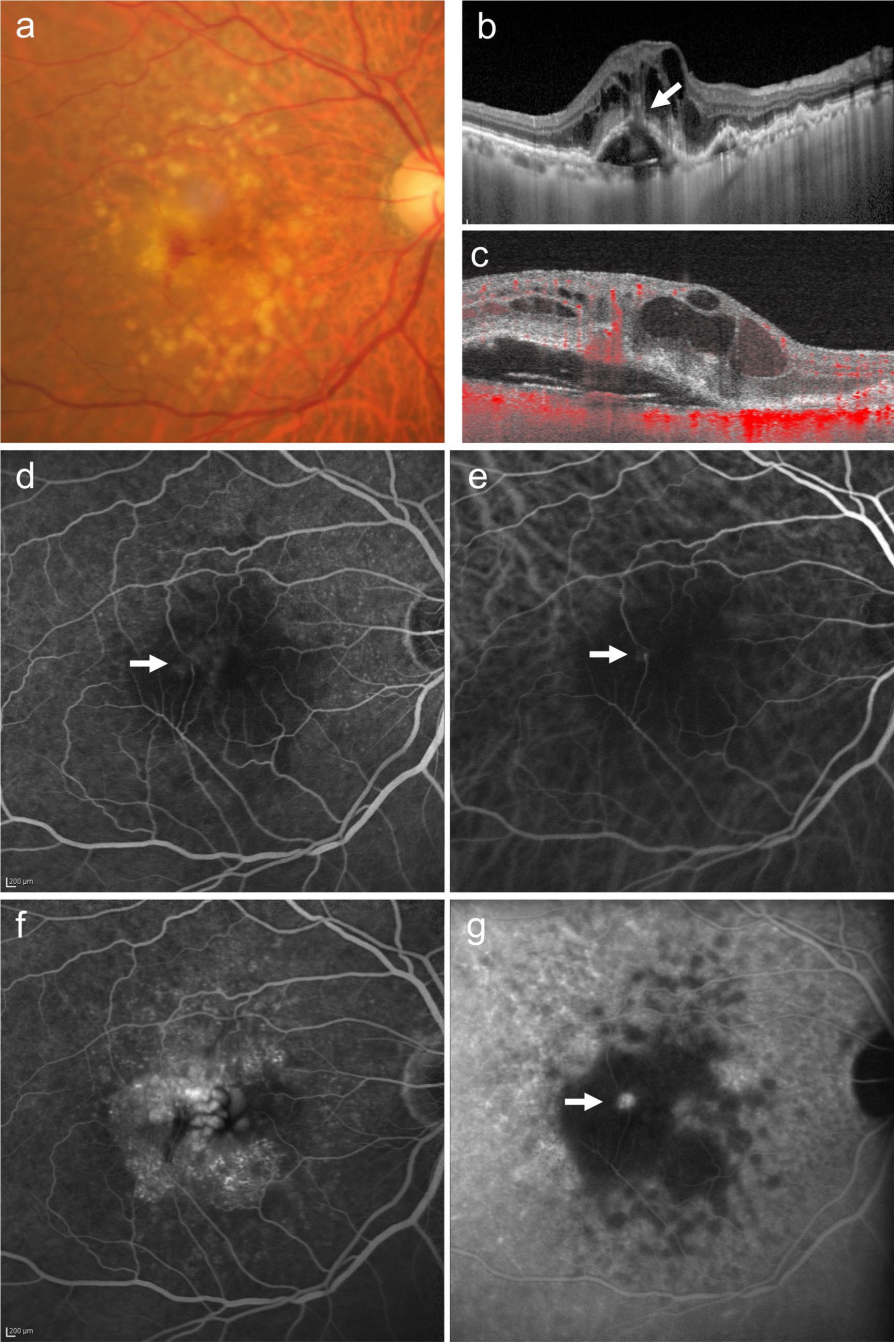
Type 3 MNV. **a** Fundus photo shows soft drusen and intraretinal hemorrhage. **b** OCT shows macular edema and a serous PED with a bump sign (arrow). **c** OCTA shows continuous blood flow signals (red) from the superficial retina to the bump sign, indicating intraretinal neovascularization. **d, e** Early-phase FA (d) and ICGA (e) show anastomosis between superficial retinal vessels and punctate hyperfluorescence (arrow). Scale bar, 200 μm. **f, g** Late-phase FA (f) shows progressive leakage, and ICGA (g) shows a hot spot (arrow). Scale bar, 200 μm

Type 3 MNV is difficult to detect by ophthalmoscopy or fundus photography. When intraretinal hemorrhage is observed in patients with numerous soft drusen bilaterally, OCT, OCTA, FA, and/or ICGA should be used for confirmation of type 3 MNV (Fig. 7). Cystoid macular edema is an early sign on OCT, and PED develops as the disease progresses. Type 3 MNV is characterized by a bump sign, where disruption occurs at the PED site (Fig. 7b). On OCTA B-scan images, intraretinal neovascularization is connected to superficial retinal vessels, extending into the subretinal space or under the sub-RPE space through the RPE. FA and ICGA demonstrate MNV anastomosis with retinal vessels, with late-phase FA demonstrating progressive leakage, and ICGA revealing a hotspot.

### Diagnosis of initial pathological findings

#### Drusen

**Fig. 8:**
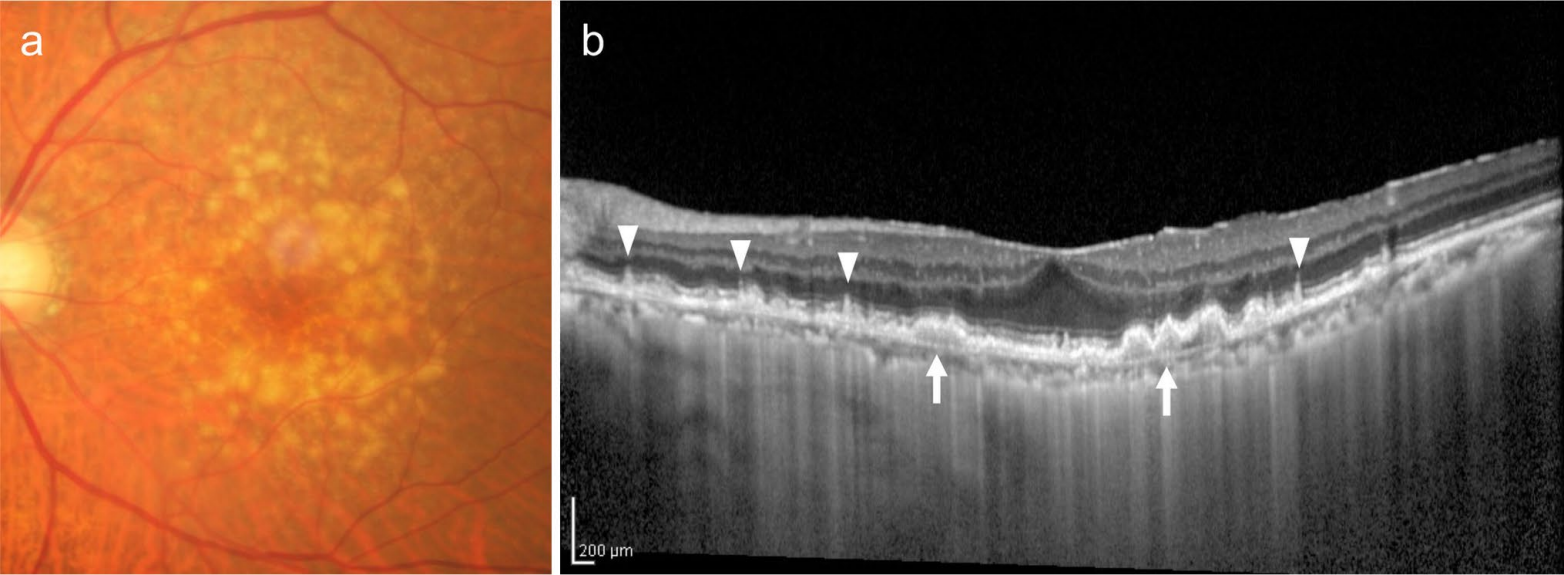
Drusen. **a** Fundus photo shows multiple drusen. **b** OCT shows large soft drusen (arrow) and subretinal drusenoid deposits (arrowhead). Scale bar, 200 μm

Drusen and drusenoid PED, which are confluent drusen deposits, can be detected through fundus examination. Distinguishing these similar findings is easier with OCT (Fig. 8). Differentiation from serous PED is based on the reflectivity of the contents. Drusen or drusenoid PEDs exhibit moderate reflectivity, whereas serous PEDs exhibit low reflectivity. Additionally, drusen are located beneath the RPE. In contrast, subretinal drusenoid deposits are located on the RPE.

#### Pachychoroid

**Fig. 9:**
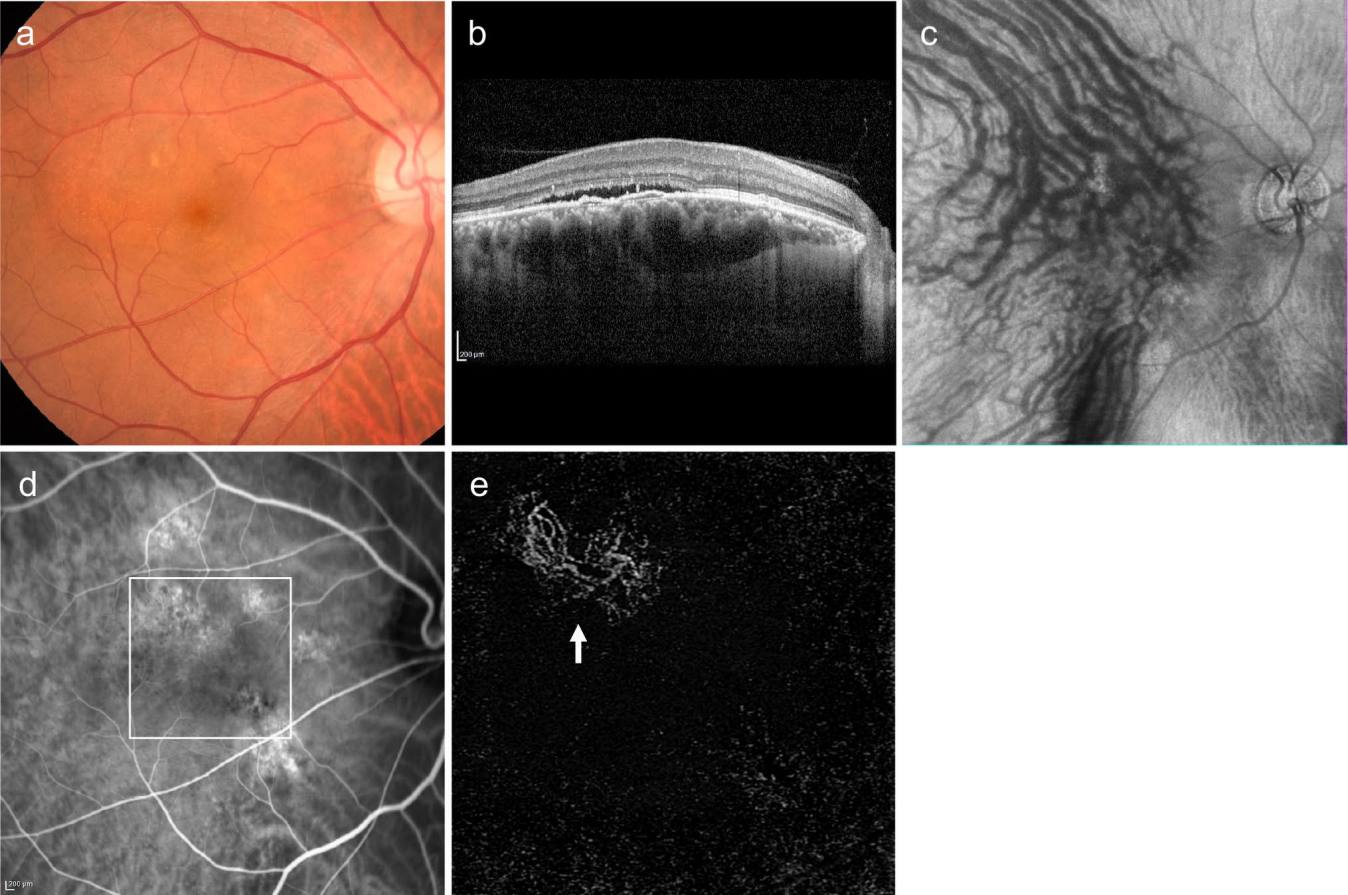
Pachychoroid neovasculopathy. **a** Fundus photo shows serous retinal detachment and attenuated fundus tessellation. **b** OCT shows dilated large choroidal vessels, thinning of the choroidal inner layer, subretinal fluid, and a shallow elevation of the RPE with a double layer sign. Scale bar, 200 μm. **c** The en face OCT image shows dilated large choroidal vessels. **d** Mid-phase ICGA shows choroidal vascular hyperpermeability, but MNV is difficult to determine. Scale bar, 200 μm. e: The en face OCTA image from the RPE to the choriocapillaris, corresponding to the area indicated by the white square in (d) shows MNV (arrow)

Choroid thickening often reduces the visibility of the choroidal vasculature on ophthalmoscopy and fundus photography. On OCT, choroidal vessels in Haller’s layer are dilated, while the choriocapillaris and Sattler’s layers are thinned. Large dilated choroidal vessels can be identified via *en face* OCT and ICGA. Choroidal vascular hyperpermeability is often observed from the mid to late phases of ICGA (Fig. 9). Large drusen, known as pachydrusen, are occasionally observed, although their pathological significance is considered minimal [29].

#### RPE abnormalities

Pigmentary abnormalities of the RPE manifest as pigment loss, hyperpigmentation, or pigmentary mottling on ophthalmoscopy and fundus photography. FAF demonstrates hypo- or hyperfluorescence, depending on the degree and state of RPE damage. FA demonstrates hyperfluorescence due to window defects in areas of pigment loss, hypofluorescence due to fluorescence blockage in hyperpigmented areas, and mixed hypo- and hyperfluorescence in pigmentary mottling areas. Serous PED without MNV presents as a well-defined dome-shaped elevation with a yellow-brown border on ophthalmoscopy and fundus photography. Dye pooling on FA corresponds to PED.

### Assessment of disease activity

MNV exudative changes indicate that the disease-status is active. The absence of exudative changes indicates inactive status. In addition to fundus examinations, disease activity is now usually determined noninvasively using OCT. Previously, the extent of fluorescein leakage was assessed on FA.

**Fig. 10:**
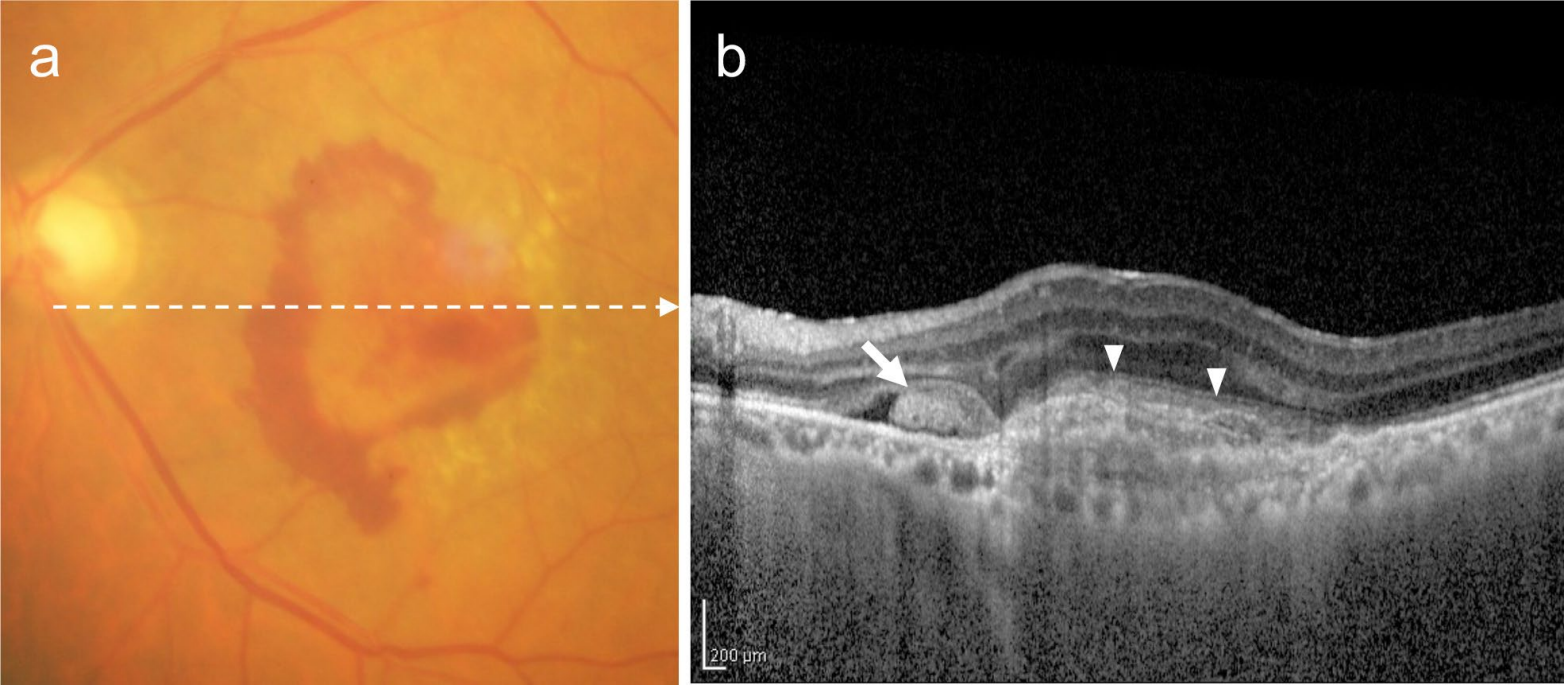
Type 2 MNV. **a** Fundus photo shows a gray-white lesion with subretinal hemorrhage and fibrinous exudates. **b** OCT corresponding to the dashed arrow in (a) shows subretinal hemorrhage (arrow) as well as fibrin and MNV (arrowhead) observed as subretinal hyperreflective materials. Scale bar, 200 μm

Exudative changes include retinal edema with intraretinal cysts (intraretinal fluid [IRF]), serous retinal detachment with subretinal fluid (SRF), serous PED (sub-RPE fluid), intraretinal hemorrhage, subretinal hemorrhage, sub-RPE hemorrhage (hemorrhagic PED), fibrin, exudates, etc. OCT is a useful tool for fluid detection. Intraretinal hemorrhage, subretinal hemorrhage, and sub-RPE hemorrhage (hemorrhagic PED) can be observed using ophthalmoscopy and fundus photography, while OCT is used to confirm their location. The subretinal hemorrhage appears as subretinal hyperreflective material (SHRM) on OCT (Figs 5 and 10). Severe hemorrhage can lead to submacular and vitreous hemorrhage. Fibrin is also observed as SHRM on OCT and subretinal or intraretinal hard exudates are observed as hyperreflective foci.

Serous PED can be relatively easy to detect based on its appearance as a well-defined, dome-shaped elevation with a yellow-brown border, as observed in ophthalmoscopic findings and fundus photographs (Fig. 4). The differentiation between serous and hemorrhagic PED depends on the content; if the exudate is transparent, serous PED is considered. In contrast, if the exudate is red-black and hemorrhagic, or white with organized blood clots, it is classified as hemorrhagic PED.

**Fig. 11:**
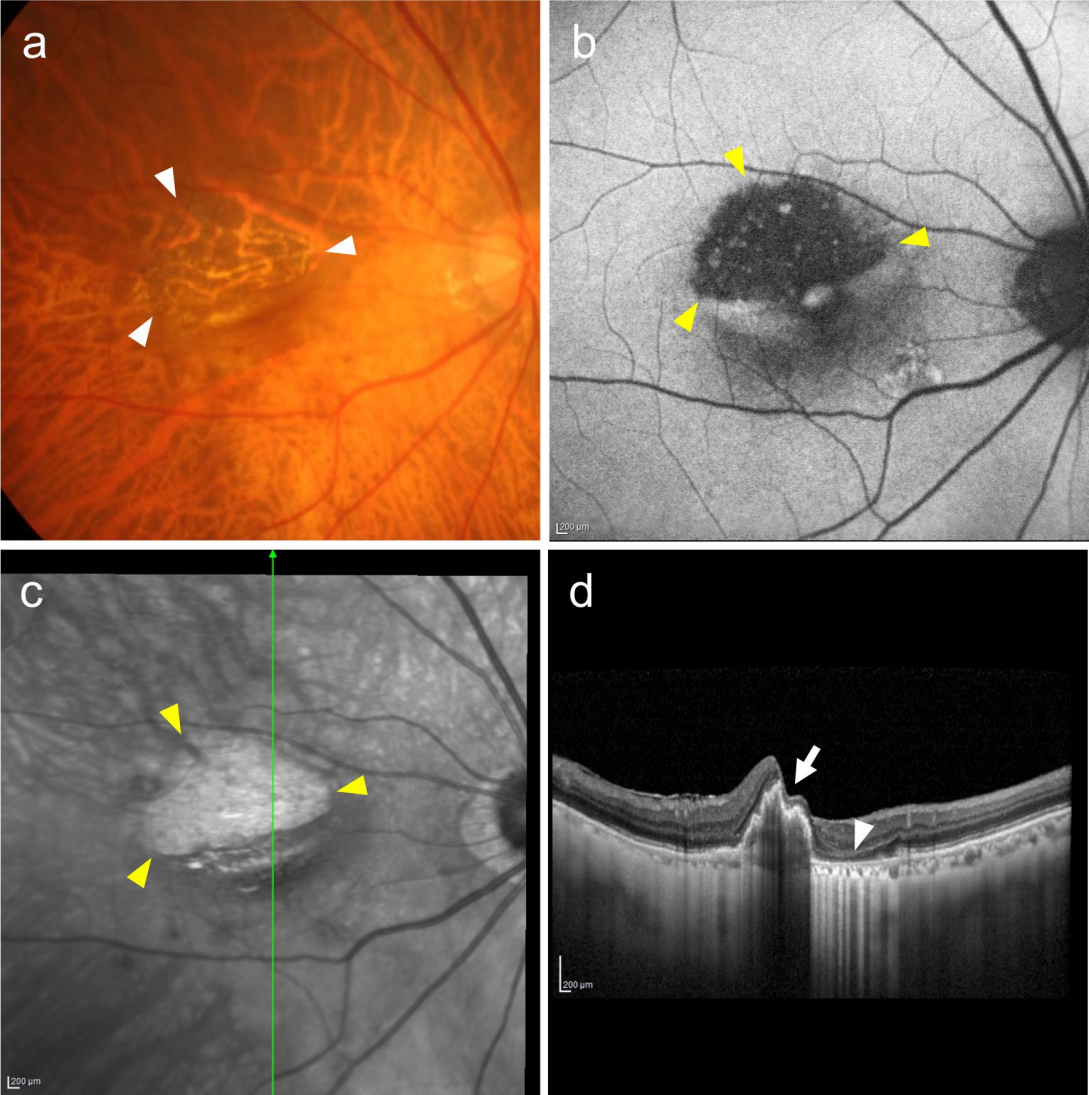
Retinal pigment epithelium tear. **a** Fundus photo shows choroid visible through the RPE defect (arrowhead). **b** Fundus autofluorescence shows hypofluorescence, indicating the RPE defect (arrowhead). Scale bar, 200 μm. **c** Near-infrared image shows the RPE tear area as a hyperreflective area (arrowhead). Scale bar, 200 μm. **d** OCT corresponding to the green arrow in (c) shows RPE shrinkage (rolling, arrow) and RPE defect (arrowhead). Scale bar, 200 μm

PED can cause RPE tears, either spontaneously or after treatment. These RPE tears can be detected on ophthalmoscopic findings and fundus photographs. However, they become more apparent on FAF because of hypofluorescence at the site of RPE loss (Fig. 11).

**Fig. 12:**
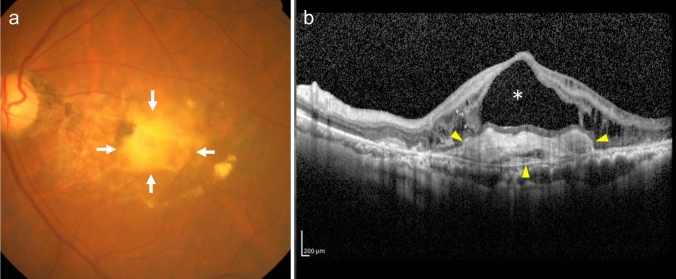
Fibrotic scar with cystoid macular degeneration. **a** Fundus photo shows a white to yellow-white fibrotic scar (arrow). **b** OCT shows a hyperreflective area under the retina indicating fibrotic scar and cystoid spaces (*) within the retina. Scale bar, 200 μm

The presence of fibrous scars in end-stage AMD can be confirmed using fundus examination and OCT. Cystoid cavities associated with cystoid macular degeneration can sometimes be observed on fundus examination, but are more easily confirmed with OCT (Fig. 12). In the FA of cystoid macular degeneration, there is no significant leakage of the dye, but only dye accumulation within the cysts. Additionally, cystoid macular degeneration is often accompanied by outer retinal atrophy and fibrous scars. These features distinguish cystoid macular degeneration from cystoid macular edema. Cystoid cavities associated with cystoid macular degeneration are not used to evaluate the disease activity. Notably, these cavities often do not resolve with treatment, and even if they do, visual recovery may be limited.

## Treatment and management

A treatment and management flowchart is shown in Fig. 1. Based on current evidence, primarily from multicenter prospective studies, these treatment guidelines were developed for the management of neovascular AMD in Asian patients, including Japanese patients, while also allowing for the selection of treatment methods tailored to individual cases. The following sections provide detailed explanations of the various treatment and management options.

### Lifestyle guidance

#### Smoking cessation

Smoking is a modifiable risk factor, and active smoking cessation guidance to patients is recommended. The relationship between smoking and AMD is reported in Japanese epidemiological studies, such as the Funagata [30], Hisayama [31], and Nagahama studies [32]. The Hisayama study, an important longitudinal study with a nine-year follow-up period, reports that smoking increases the incidence of late-stage AMD by four times.

#### Improvement of dietary habits

A diet rich in long-chain omega-3 polyunsaturated fatty acids, minerals (e.g., zinc, copper, etc.), vitamin C, vitamin E, antioxidant carotenoids (e.g., beta-carotene, lutein, zeaxanthin, etc.), and low levels of saturated or monounsaturated fatty acids reduces the risk of developing AMD, as well as the risk of progression from intermediate to late AMD [33, 34]. Long-chain omega-3 polyunsaturated fatty acids are found abundantly in fish. Fruits and vegetables, especially green and yellow vegetables, contain high levels of antioxidant vitamins, such as vitamin C and carotenoids. A diet that restricts the intake of saturated or monounsaturated fatty acids and increases fish, fruits, and vegetable consumption is recommended at all AMD stages [35]. At the same time it is important to note, that due to the limited number of nutritional epidemiological studies on AMD involving Japanese subjects, there is a possibility that the results reported in Western populations may not be applicable to Japanese individuals.

#### Supplements

The AREDS conducted in the United States report that the intake of supplements containing antioxidant vitamins, beta-carotene, and zinc reduced the progression to late AMD in eyes with intermediate AMD (characterized by multiple medium-sized drusen, at least one large drusen, or geographic atrophy without foveal involvement), or in eyes already affected by late AMD in the fellow eye [36]. As beta-carotene intake in smokers is associated with an increased risk of lung cancer, AREDS2 recommends replacing beta-carotene with lutein/zeaxanthin and reducing zinc intake from 80 mg to 25 mg. Moreover, AREDS2 demonstrates no difference in preventive efficacy following these alterations (Table 3) [37, 38]. Current evidence does not support the hypothesis that supplements can prevent the progression from early to intermediate AMD. Furthermore, the excessive intake of vitamins and zinc may have side effects; therefore, caution is necessary when using these supplements.

**Table 3:** Supplementation in the AREDS 2

Components	Daily intake
Vitamin C Vitamin E Lutein/zeaxanthin Zinc oxide Cupric oxide	500 mg 400 IU 10 mg/2 mg 25 mg 2 mg

Available evidence is based on studies conducted in the United States and has not been validated in a Japanese population. However, a small-scale study involving Japanese participants reports that patients who did not take supplements demonstrated a higher risk of progression to neovascular AMD relative to those who took supplements [39].

### Pharmaceutical treatment

#### Intravitreal injection of anti-VEGF drugs

#### Drug types

The first-line treatment for neovascular AMD is the intravitreal injection of anti-VEGF drugs [40]. In Japan, the currently available anti-VEGF drugs for neovascular AMD include ranibizumab (Lucentis^®^ and ranibizumab biosimilar), aflibercept (Eylea^®^ 2 mg and 8 mg), brolucizumab (Beovu^®^), and faricimab (Vabysmo^®^), an antibody against VEGF and angiopoietin-2 (Table 4). Representative clinical trials of these drugs (e.g., MARINA study [41]; ANCHOR study [42]; VIEW 1/2 study [43]; HAWK/HARRIER study [44]; TENAYA/LUCERNE study [45]; PULSAR study [46]) demonstrate improved visual acuity. Although intraocular inflammation associated with anti-VEGF drugs can occur with all these agents, brolucizumab injection is associated with higher incidences of retinal vasculitis and retinal vascular occlusion, which necessitate early detection and management of intraocular inflammation [47, 48].

**Table 4:** Anti-vascular endothelial agents and clinical trials for neovascular age-related macular degeneration

Drugs	Clinical Trials	Observation periods	Results
Ranibizumab	MARINA study	24 months	Visual acuity loss fewer than 15 letters was reported 62% of sham arm and 95% in treated arm with monthly ranibizumab injections. Visual acuity decreased by 14.9 letters in sham arm and improved by 6.6 letters in ranibizumab-treated arm.
	ANCHOR study	12 months	Visual acuity loss fewer than 15 letters was reported 64.3% in PDT arm and 96% in treated arm with ranibizumab monthly injections. Visual acuity decreased by 9.5 letters in PDT arm and improved by 11.3 letters in ranibizumab-treated arm.
Aflibercept 2 mg	VIEW 1/2 study	52 weeks	Aflibercept (3 loading doses + every 8 weeks) was non-inferior to monthly ranibizumab in maintaining visual acuity.
Brolucizumab	HAWK/HARRIER study	48 weeks	Brlolucizumab (3 loading doses + every 8/12 weeks) was non-inferior to aflibercept (3 loading doses + every 8 weeks) in vision improvement. Brolucizumab arm showed a significant reduction in central retinal thickness compared to aflibercept arm.
Faricimab	TENAYA/LUCERNE study	48 weeks	Faricimab (4 loading doses + every 8/12/16 weeks) was non-inferior to aflibercept (3 loading doses + every 8 weeks) in vision improvement.
Aflibercept 8 mg	PULSAR study	48 weeks	Aflibercept 8 mg (3 loading doses + every 12/16 weeks) was non-inferior to aflibercept 2 mt (3 loading doses + every 8 weeks) in vision improvement.

The number of letters was determined using the Early Treatment Diabetic Retinopathy Study (ETDRS) visual acuity chart.

**Fig. 13:**
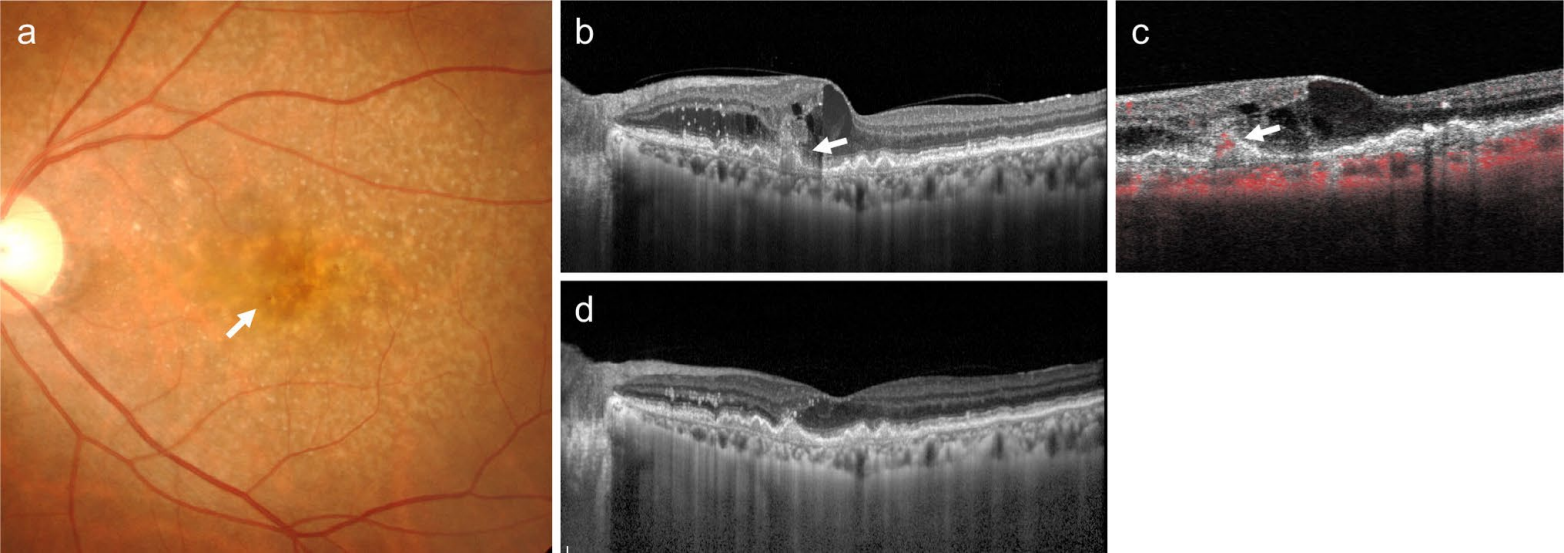
Type 3 MNV treated with anti-VEGF drugs. **a** Pre-treatment fundus photo shows punctate intraretinal hemorrhage (arrow). **b** OCT shows retinal edema and RPE disruption (arrow). **c** OCTA corresponding to B shows blood flow signals (red) indicating intraretinal neovascularization from the retina to the RPE disruption site (arrow). **d** One month after anti-VEGF treatment, the intraretinal edema has resolved

Across these clinical trials, intravitreal injections of anti-VEGF drugs have demonstrated efficacy in improving visual acuity regardless of the neovascular AMD subtype (Fig. 13). Clinical trials involving PCV, which is more common among Asians, including the Japanese population, report favorable treatment outcomes with aflibercept and brolucizumab [47, 49].

#### Treatment regimens

The loading phase in anti-VEGF therapy refers to the period during which monthly injections (typically administered consecutively three or four times, depending on the drug) are administered to achieve visual acuity improvement. The maintenance phase refers to the subsequent period aimed at stabilizing visual acuity.

In clinical trials, a fixed dosing regimen in which the interval between injections is predetermined is predominantly used during the maintenance phase. In contrast, in clinical practice, a pro re nata (PRN) regimen, in which injections are administered based on the disease activity observed during monthly follow-ups has become common. However, studies of ranibizumab, such as CATT and HARBOR, demonstrate that PRN treatment following a loading phase resulted in decreased visual acuity over two years compared with monthly injections [50, 51]. Additionally, long-term PRN treatment in clinical settings has not been effective in maintaining improved visual acuity, instead, deterioration often occurred [52, 53]. In contrast, the treat-and-extend regimen, which adjusts the intervals between injections based on disease activity after the loading phase, has demonstrated sustained visual acuity improvement comparable to monthly injections of ranibizumab in the TREX-AMD study [54]. The ALTAIR study compared the treat-and-extend regimen of aflibercept, adjusted every 2 or 4 weeks, in Japanese patients with neovascular AMD, and reports improved visual acuity and retinal thickness at 96 weeks in both groups [55]. A Japanese prospective multicenter study using a one-month treat-and-extend regimen that involved patients with good visual acuity, also demonstrates improvement in visual acuity and a decrease in central retinal thickness over two years [56]. Systematic reviews and meta-analyses show that the treat-and-extend regimen provides visual acuity improvements similar to the fixed-dosing regimen over two years [57]. Additionally, treat-and-extend regimens provide significantly better visual outcomes than PRN regimens, although more frequent injections than the PRN regimen are required [57]. As yet, there is no consensus on the duration of treat-and-extend regimens. Furthermore, the choice of maintenance-phase dosing should be made flexibly, considering the patient's social circumstances and the condition of the fellow eye.

#### Assessment of disease activity during treatment

Disease activity in neovascular AMD refers to exudative changes (e.g., fluid, fibrin, hemorrhage, etc.) arising from MNV. Activity can be assessed using OCT, which non-invasively detects fluid accumulation around the MNV and differentiates IRF, SRF, and sub-RPE fluids. Additionally, exudations including subretinal hemorrhage and fibrin, have been observed in SHRM. Importantly, the entire macula or lesion should be scanned to accurately assess fluid and SHRM, as the site of high disease activity in MNV, particularly polypoidal lesions in PCV or intraretinal neovascularization in type 3 MNV, does not necessarily involve the fovea.

#### Switching anti-VEGF drugs

If treatment response is inadequate (i.e., resistant cases) or decreases over time (e.g., acquired tolerance), switching to another drug may be effective. Drug switching may be an option considering the treatment burden.

#### Treatment for end-stage AMD

As AMD progresses, fibrous scars, cystoid macular degeneration, and atrophic changes may occur and these features can worsen patients’ vision (Fig. 12). For patients with significantly reduced visual acuity, with low disease activity, further treatments are less helpful. Instead, such patients should be considered for observational management.

### Photodynamic therapy (PDT)

**Fig. 14:**
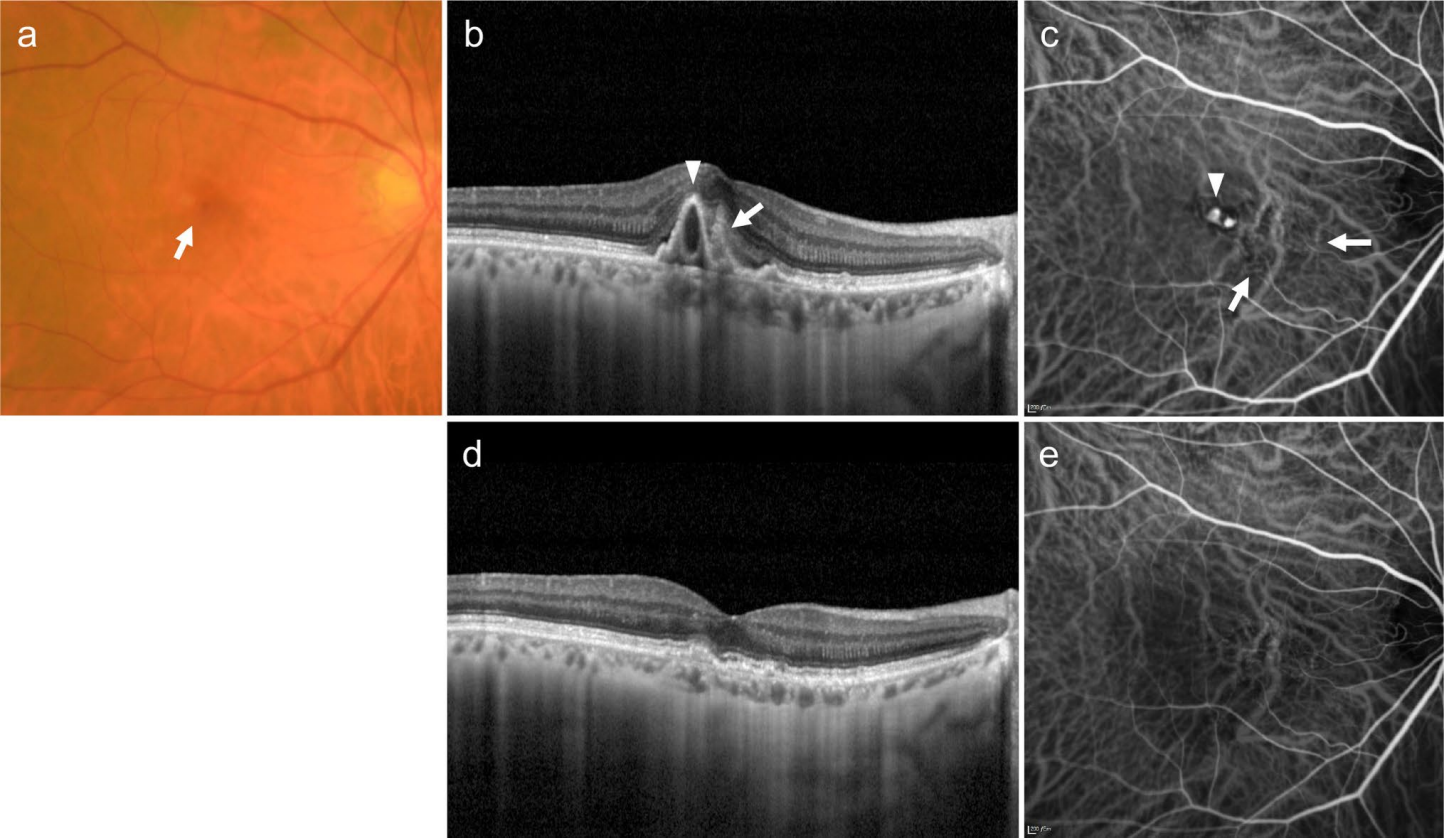
Polypoidal choroidal vasculopathy with polypoidal lesions regressed following PDT combined with anti-VEGF drug. **a** Pre-treatment fundus photo shows orange-red lesions at the fovea (arrow). **b** Pre-treatment OCT shows subretinal hyperreflective material (arrow) and protruded RPE elevation with hyporeflective spaces within (arrowhead). **c** ICGA shows type 1 MNV (arrow) and nodular polypoidal lesions (arrowhead). Scale bar, 200 μm. **d, e** After PDT combined with anti-VEGF drug, OCT shows the resolution of the protruded RPE elevation (d), and ICGA confirms the regression of polypoidal lesions (e). Scale bar, 200 μm

Currently, to improve safety and visual acuity when performing PDT for neovascular AMD, anti-VEGF drugs are usually also used [58–61]. The combination of anti-VEGF drugs and PDT remains one treatment option for PCV and is effective in improving visual acuity and leads to complete polyp regression (Fig. 14) [61]. PDT that targets areas of choroidal vascular hyperpermeability for PNV has also been attempted [62–67]. Further discussion regarding long-term effects and indications of PDT targeting choroidal vascular hyperpermeability is needed. PDT may be a potential treatment option for neovascular AMD resistant to anti-VEGF drugs.

Long-term PDT may exacerbate macular atrophy; therefore, it should be avoided in patients with a thin choroid or preexisting macular atrophy. Notably, PDT is currently not recommended for type 3 MNV.

### Other treatments

#### Laser photocoagulation

There are reports that exudative changes in the entire type 2 MNV lesions or the PCV lesions, including polypoidal lesions and the associated abnormal vascular network are suppressed by laser photocoagulation [68, 69]. However, laser photocoagulation irreversibly damages the outer retina and RPE, making it unsuitable for treating MNV close to the fovea.

#### Submacular hemorrhage displacement

**Fig. 15:**
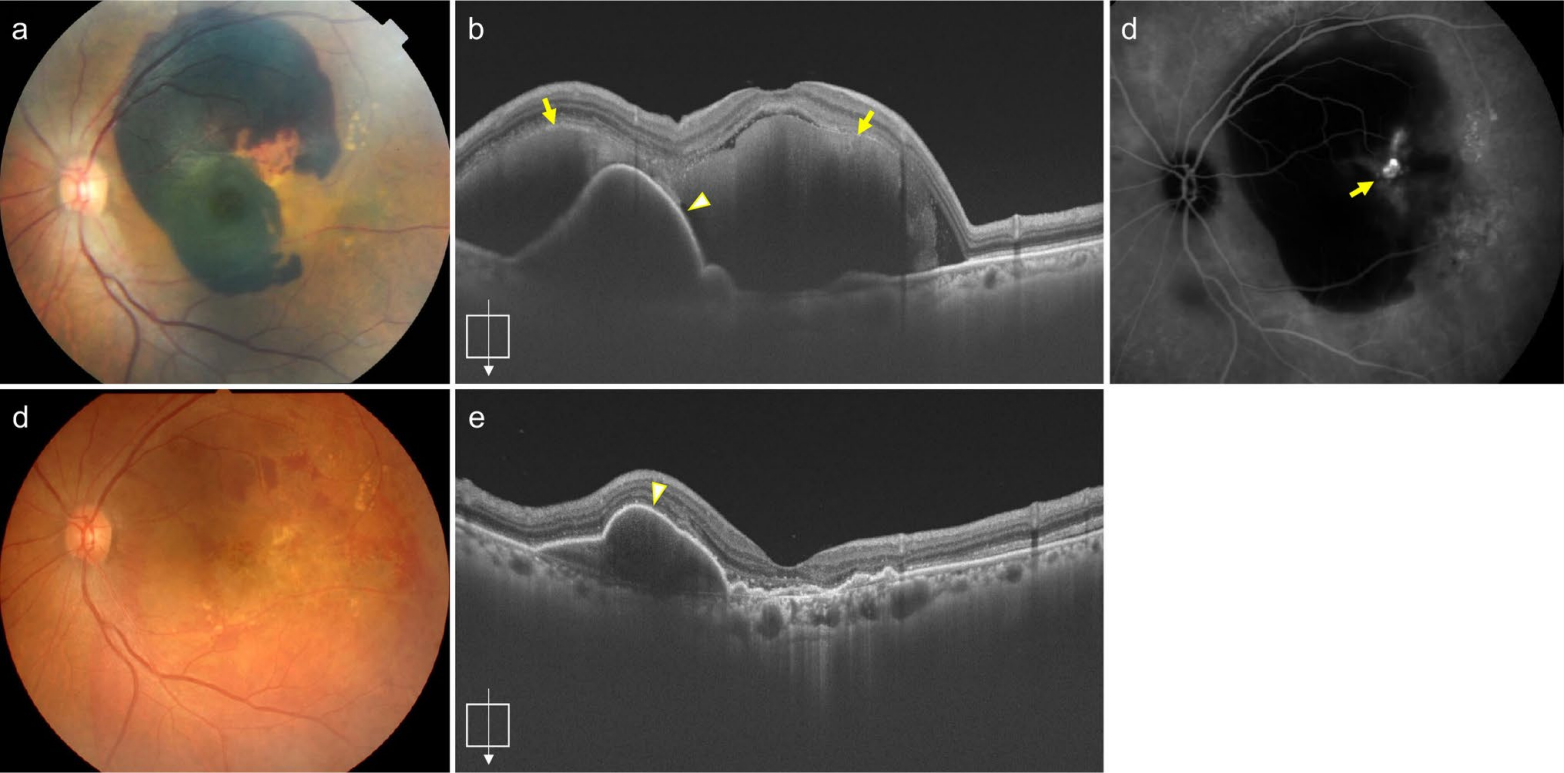
Displacement of submacular hemorrhage by intravitreal gas injection. **a** Fundus photo shows massive subretinal hemorrhage. **b** OCT shows hyperreflective subretinal hemorrhage (arrow) and PED (arrowhead). **c** ICGA shows a nodular hyperfluorescence indicative of polypoidal lesions (arrow), but the overall extent of MNV is unclear due to fluorescence blockage by hemorrhage. **d** The submacular hemorrhage is displaced from the macula following intravitreal gas injection. **e** OCT shows the resolution of submacular hemorrhage, although PED remains (arrowhead)

Neovascular AMD can cause massive submacular hemorrhage, leading to a sudden and significant decline in visual acuity (Fig. 5). Damage to the outer retina due to submacular hemorrhage causes irreversible vision loss. If detected early, visual improvements can be achieved by displacing the submacular hemorrhage (Fig. 15). Submacular hemorrhage displacement may be performed using intravitreal gas injection or vitrectomy [70, 71]. During these procedures, intravitreal injections of anti-VEGF drugs or tissue plasminogen activator (tPA, off-label use) may be used in combination. Further discussions on these indications are warranted [72–74].

#### Sub-Tenon injection of triamcinolone acetonide

Sub-Tenon injections of triamcinolone acetonide may be used to treat intraocular inflammation associated with neovascular AMD treatment [75, 76].

### Long-term management and low vision care

Currently, it is impossible to completely cure neovascular AMD. Without appropriate treatment and long-term management, irreversible vision loss is probable. Even when the activity of MNV temporarily subsides, it may recur over a long period, and repeated exudation can result in atrophic changes or fibrotic scarring leading to significant vision loss. Moreover, MNV frequently develops in the fellow eye, therefore, long-term management is necessary to avoid irreversible vision loss bilaterally. However, it is important to choose a sustainable management method for the patient, considering the disease condition and patient burden. Active engagement in low vision care is recommended for patients with severely impaired visual function [77].

## Conclusion

These new clinical guidelines for neovascular AMD include current understanding of disease staging, diagnostic criteria and treatment methods. To stay in line with the latest knowledge, these guidelines should be reviewed and updated periodically.
